# Corneal Transplantation in Disease Affecting Only One Eye: Does It Make a Difference to Habitual Binocular Viewing?

**DOI:** 10.1371/journal.pone.0150118

**Published:** 2016-03-03

**Authors:** Praveen K. Bandela, PremNandhini Satgunam, Prashant Garg, Shrikant R. Bharadwaj

**Affiliations:** 1 Prof. Brien Holden Eye Research Centre, Hyderabad Eye Research Foundation, L V Prasad Eye Institute, Hyderabad– 500034 Telangana, India; 2 Bausch and Lomb School of Optometry, L V Prasad Eye Institute, Hyderabad– 500034 Telangana, India; 3 Cornea and Anterior Segment Services, L V Prasad Eye Institute, Hyderabad– 500034 Telangana, India; University of Illinois at Chicago, UNITED STATES

## Abstract

**Background:**

Clarity of the transplanted tissue and restoration of visual acuity are the two primary metrics for evaluating the success of corneal transplantation. Participation of the transplanted eye in habitual binocular viewing is seldom evaluated post-operatively. In unilateral corneal disease, the transplanted eye may remain functionally inactive during binocular viewing due to its suboptimal visual acuity and poor image quality, vis-à-vis the healthy fellow eye.

**Methods and Findings:**

This study prospectively quantified the contribution of the transplanted eye towards habitual binocular viewing in 25 cases with unilateral transplants [40yrs (IQR: 32–42yrs) and 25 age-matched controls [30yrs (25–37yrs)]. Binocular functions including visual field extent, high-contrast logMAR acuity, suppression threshold and stereoacuity were assessed using standard psychophysical paradigms. Optical quality of all eyes was determined from wavefront aberrometry measurements.

Binocular visual field expanded by a median 21% (IQR: 18–29%) compared to the monocular field of cases and controls (p = 0.63). Binocular logMAR acuity [0.0 (0.0–0.0)] almost always followed the fellow eye’s acuity [0.00 (0.00 –-0.02)] (r = 0.82), independent of the transplanted eye’s acuity [0.34 (0.2–0.5)] (r = 0.04). Suppression threshold and stereoacuity were poorer in cases [30.1% (13.5–44.3%); 620.8arc sec (370.3–988.2arc sec)] than in controls [79% (63.5–100%); 16.3arc sec (10.6–25.5arc sec)] (p<0.001). Higher-order wavefront aberrations of the transplanted eye [0.34μ (0.21–0.51μ)] were higher than the fellow eye [0.07μ (0.05–0.11μ)] (p<0.001) and their reduction with RGP contact lenses [0.09μ (0.08–0.12μ)] significantly improved the suppression threshold [65% (50–72%)] and stereoacuity [56.6arc sec (47.7–181.6arc sec)] (p<0.001).

**Conclusions:**

In unilateral corneal disease, the transplanted eye does participate in gross binocular viewing but offers limited support to fine levels of binocularity. Improvement in the transplanted eye’s optics enhances its participation in binocular viewing. Current metrics of this treatment success can expand to include measures of binocularity to assess the functional benefit of the transplantation process in unilateral corneal disease.

## Introduction

Corneal opacity is a leading cause of blindness and vision impairment worldwide[1,2]. Infections or trauma to one eye are the most common causes of corneal opacity in developing nations and they are responsible for about 1.5 to 2 million new cases every year [3]. Vision restoration following corneal blindness involves surgically replacing the diseased tissue with a healthy and clear cornea from a donor [3]. This procedure–penetrating keratoplasty–remains the gold-standard treatment even as newer surgical procedures are evolving to transplant only parts of diseased cornea for vision restoration (e.g. Descemet’s Stripping Endothelial Keratoplasty [DSEK] for corneal endothelial disease[4]).

The success of penetrating keratoplasty is to-date evaluated on the post-operative clarity of the transplanted tissue and the restoration of monocular high-contrast visual acuity in the transplanted eye [5,6]. Successful transplantation also improves the quality of life of these individuals, with the improvement being understandably greater in bilateral than unilateral transplants [7,8]. While these are perfectly acceptable metrics for evaluating the outcomes of transplantation, they do not measure the transplanted eye’s participation in habitual viewing. Habitual viewing is a binocular process and many visual functions (e.g. fine depth perception) are crucially dependent on the matching of corresponding features in the two eyes [9]. Such binocular functions that require active participation of the transplanted eye are seldom evaluated post-operatively.

Despite anatomical clarity, the transplanted eye may be optically severely degraded due to irregular astigmatism (~3.2D after 1-yearof surgery [10,11]) and higher-order wavefront aberrations (HOAs; about six times greater in the transplanted eye than in age-matched controls [12,13,14]) that arise post-operatively. Individuals undergoing transplantation for unilateral corneal disease may therefore experience a large interocular difference in image quality, with the transplanted eye’s image quality being significantly inferior to that of the healthy fellow eye. Previous studies examining the impact of induced interocular differences in image quality all report that binocular visual functions such as stereoacuity [15] and binocular summation [16] may be significantly deteriorated while other spatial visual functions may be biased towards the eye providing better image quality (e.g. binocular logMAR acuity [17,18,19]). Overall, the eye experiencing the poor image quality may not actively participate in the binocular viewing process. While some of the interocular differences in image quality following unilateral corneal transplantation may be corrected with sphero-cylindrical spectacles, those arising from HOA’s remain uncorrected with conventional spectacles. Given this, to what extent does the transplanted eye participate in habitual binocular viewing of those undergoing unilateral corneal transplantation? Also, how does the optical quality of the transplanted eye influence its participation in functional binocular vision and does an improvement in the eye’s optical quality produce a commensurate improvement in binocular vision? Overall, what is the functional utility of transplantation in unilateral corneal disease? Answers to these questions are unknown even while they have important implications for utilization of donor corneas available for transplantation and for managing patient’s expectations about functional vision restoration post-operatively. To the best of our knowledge, questions of similar nature have been addressed by only one study in the past for individuals undergoing bilateral corneal transplantation [20].

## Materials and Methods

Two experiments were conducted in this study. The first experiment evaluated the participation of transplanted eye in a range of binocular visual functions in 25 patients (cases) [median age: 40yrs (Inter Quartile Range (IQR): 32–42yrs)] and compared these with 25 age-matched controls with normal binocular vision [30yrs (25–37yrs)] (Table 1). Cases underwent corneal transplantation for the following reasons: microbial keratitis (n = 17), corneal scar from trauma (n = 7) and pseudophakic bullous keratopathy (n = 1). All the transplanted donor corneas were obtained from L V Prasad Eye Institute’s Ramayamma International Eye bank, Hyderabad. Eye bank procedures including consent from the relatives of the deceased donors retrieval and storage of the donor corneas were done as per the standard operating procedures of the eye bank. A second experiment determined the impact of manipulating the transplanted eye’s optics using rigid gas permeable (RGP) contact lenses on binocular visual functions in a subset of 10 cases [38yrs (32.3–40.8yrs)] that participated in the first experiment. RGP contact lenses have been used in the past for managing the post-operative refractive error in patients who undergo corneal transplantation [10,21,22,23]. While these lenses have been shown to effectively reduce irregular astigmatism, interocular difference in refractive power (anisometropia) and improve logMAR acuity, very little is known about how they impact HOA’s and sensory binocular visual performance in these patients [10,21,22,23].

**Table 1:** Demographic details of the cases and controls who participated in this study.

Variable	Cases Median (IQR)	Controls Median (IQR)	p value
**Total Number**	25	25	---
**Age (years)**	40 (32–42)	30 (25–37)	0.06
**Duration between trauma and surgery (months)**	4.06 (0.93–12.25)	NA	---
**Duration between surgery and present investigation (months)**	16.9 (9.2–33.3)	NA	---
**M (D)**	-0.75 (-3.21–0.06)	0.00D (-0.37–0.00)	0.22
**J0 (D)**	0.00 (-0.75–0.79)	0.00 (0.00–0.00)	0.91
**J45 (D)**	-0.18 (-1.55–0.36)	0.00 (0.00–0.00)	0.13

The study adhered to the tenets of the Declaration of Helsinki and was approved by the Institutional Review Board of L V Prasad Eye Institute (LVPEI), Hyderabad, India. All subjects participated after signing a written informed consent form. The inclusion criteria for enrolling cases in the study were: a) Cornea transplantation procedure in one eye only and the fellow eye being free of any ocular pathology; 2) postoperative follow-up of at least 3 months–the median (25^th^–75^th^ IQR) follow-up duration was 16.9 (9.2–33.3) months between surgery and the present investigation (Table 1); 3) Clear transplanted cornea and 4) absence of co-morbidity that would affect the visual functions of the operated eye (e.g. glaucoma, iris synechiae, etc). Cases with unstable refraction in previous follow-ups or with any manifest eye deviation that affected binocularity were excluded. Standard clinical management was followed in all cases, with no influence of the study on their care. Controls were students and staff of LVPEI with normal binocular vision.

Wavefront aberrations were measured using the Imagine Eyes irx3™ wavefront aberrometer (http://www.imagine-eyes.com/wp-content/uploads/2014/08/M-DCP-001-g-irx3-datasheet.pdf) three times in each eye for 3mm pupil diameter and averaged [24]. All measurements were made with the subject’s natural pupil size and accommodative state in order to mimic habitual viewing conditions. Subjects were fixated at optical infinity using the aberrometer’s target presentation channel during these measurements. The wavefront aberrations were then scaled to the 3mm pupil diameter for all subjects using algorithms previously developed and described for this purpose [25]. The eye’s optical quality was described in terms of the overall root mean squared deviation of the 3^rd^ to 8^th^ order wavefront aberration terms (HORMS). Wavefront aberrations could not be obtained on one case and one control due to small pupils (~2mm) and on 2 cases for unknown reasons.

Monocular and binocular visual fields, high contrast logMAR acuity, suppression threshold and stereoacuity were measured on each subject with their best-corrected sphero-cylindrical correction. For cases, performance of the fellow healthy eye was always evaluated first followed by that of the transplanted eye and then binocular performance in order to maximize task learning. The order of testing was randomized for controls. Kinetic perimetry (Humphrey Visual Field Analyzer II, Carl Zeiss, Dublin) was performed to determine the subject’s monocular and binocular visual fields [26]. Maximum of 150° of horizontal and 110° of vertical visual fields were measured in 12 discrete meridians, interpolated using a spline algorithm, and the area under this curve calculated using custom MATLAB® software. The ratio of binocular to monocular visual field area was calculated for all subjects, with a value >1 indicating an expansion of the binocular visual field relative to its monocular counterparts [27]. For cases, the ratio of binocular to healthy fellow eye’s visual field area was calculated and a value of unity indicated that the transplanted eye did not contribute to the expansion of binocular visual field. Monocular visual field area of one eye was chosen randomly in controls for this ratio calculation. Four cases could not perform the kinetic perimetry test while all controls performed this task successfully.

Monocular and binocular high-contrast (98%) logMAR acuity of all subjects was determined at 3m using COMPlog® (http://www.complog-acuity.com/) [28]. A series of 5 Sloan optotypes were randomly displayed and their angular subtense decreased using a staircase thresholding algorithm until 3 out of 5 optotypes were incorrectly identified. LogMAR acuity was recorded as the number of optotypes correctly identified at termination, with 0.02 logMAR units allotted per optotype [28]. All cases and controls performed this task successfully.

Suppression threshold was determined at 40cm with the subject’s best-corrected distance and near refractive correction using a dichoptic motion coherence threshold task designed previously for subjects with amblyopia [29,30]. The task involves detecting the direction of coherent motion of a field of ‘signal’ dots from a field of randomly moving ‘noise’ dots. A binocular threshold is first estimated as the minimum number of ‘signal’ dots required in the two eyes to detect binocular coherent motion. This value is then used to determine the magnitude of suppression by dichoptically presenting the coherently moving ‘signal’ dots to the affected eye (i.e. amblyopic eye in the previous studies [29,30] and transplanted eye in this study) and randomly moving ‘noise’ dots to the fellow eye. The contrast of ‘signal’ dots is maintained at ~100% while the contrast of ‘noise’ dots is varied in a staircase manner until coherent motion perception is abolished. This contrast threshold provides a quantitative estimate of suppression of the affected eye–lower the contrast of the ‘noise’ dots, greater is the magnitude of suppression of the affected eye [29,30]. In controls, the ‘noise’ dots were presented randomly to either eye as there was no expectation of one eye being weaker than the other (ocular dominance status of controls was not determined here). Five cases could not complete this task while all controls performed this task successfully.

Stereoacuity was measured thrice using a custom-designed Howard Dolman apparatus where subjects aligned two thin rods presented in depth against a monocular-depth-cue-free background [9,31]. One rod was moved back and forth in a motorized fashion, relative the other, until the subjects perceived both rods to be at the same depth plane. Axial separation between the two rods provided a measure of the subject’s stereoacuity [9,31]. Measurements were usually made at 3m but they were shifted to 1m if subjects were unable to perform the task at 3m. Between 1 and 3m, this set up produced disparities from 1.5 to 2263arcsec for 60mm IPD (variations in IPD of a few millimeters producing an inconsequential 5–6arc sec change in disparity values [9]). All cases and controls performed this task successfully.

All aforementioned outcome variables (except visual fields whose outcomes on cases were at par with controls–see results) were re-measured in a subset of 10 cases after 30min of adaptation to a customized RGP contact lens fitted to the transplanted eye. Subjects wore CLASSIC® or Purecon® RGP lenses, both of which are commonly available for clinical use in India. Residual refractive error obtained over the contact lenses was corrected with spectacles during data collection.

## Results

Table 1 shows the demographic details and the power vector representation of sphero-cylindrical refractive error of all controls and cases [32]. Since most outcomes variables obtained here did not follow a normal distribution (Shapiro-Wilk normality test), non-parametric statistics were used for data analyses.

**Fig 1:**
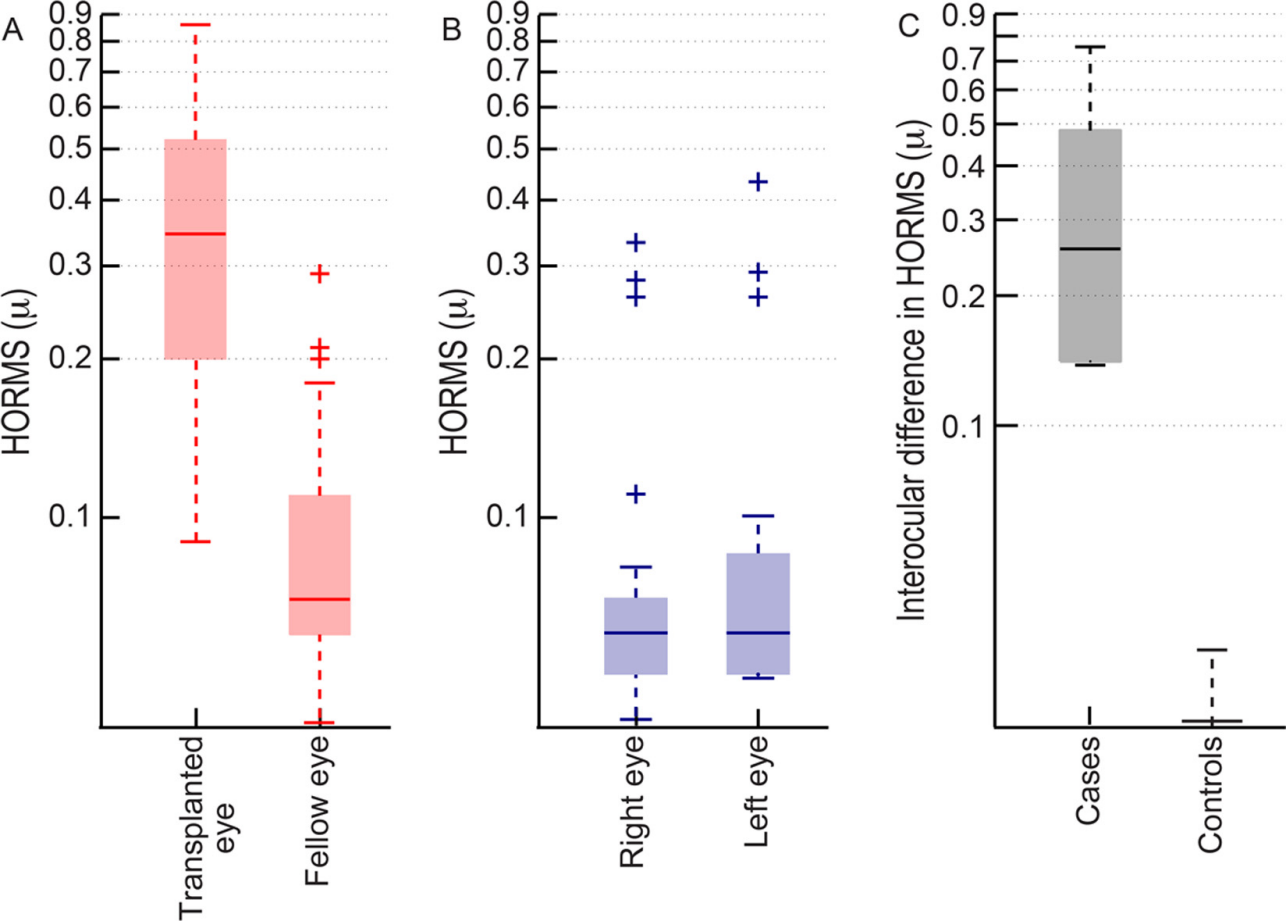
Box and Whisker plot of the RMS deviation of higher-order wavefront aberrations (HORMS) measured for 3mm pupil diameter for cases (panel A) and controls (panel B) that participated in this study. Panel C: The interocular difference in HORMS for cases and controls that participated in this study. The solid horizontal line within the box indicates median value, lower and upper edges of the box indicate the 25^th^ and 75^th^interquartile range (IQR), lower and upper whiskers show the 1^st^and 99^th^ quartiles and plus symbols indicate outliers.

HORMS of the transplanted eye [0.34μ (0.21–0.51μ)] was significantly greater than that of the fellow healthy eye [0.07μ (0.05–0.11μ)] of cases (Mann-Whitney U = 56; n = 46; p<0.001) while they were similar to each other in the right and left eyes of controls [both eyes: 0.05μ (0.05–0.08μ)] (p = 0.4) (Fig 1A). Interocular difference in HORMS was also greater in cases [0.25μ (0.14–0.48μ)] than in controls [0.00μ (-0.02–0.01μ)] (U = 37.5; n = 46; p<0.001) (Fig 1C).

**Fig 2:**
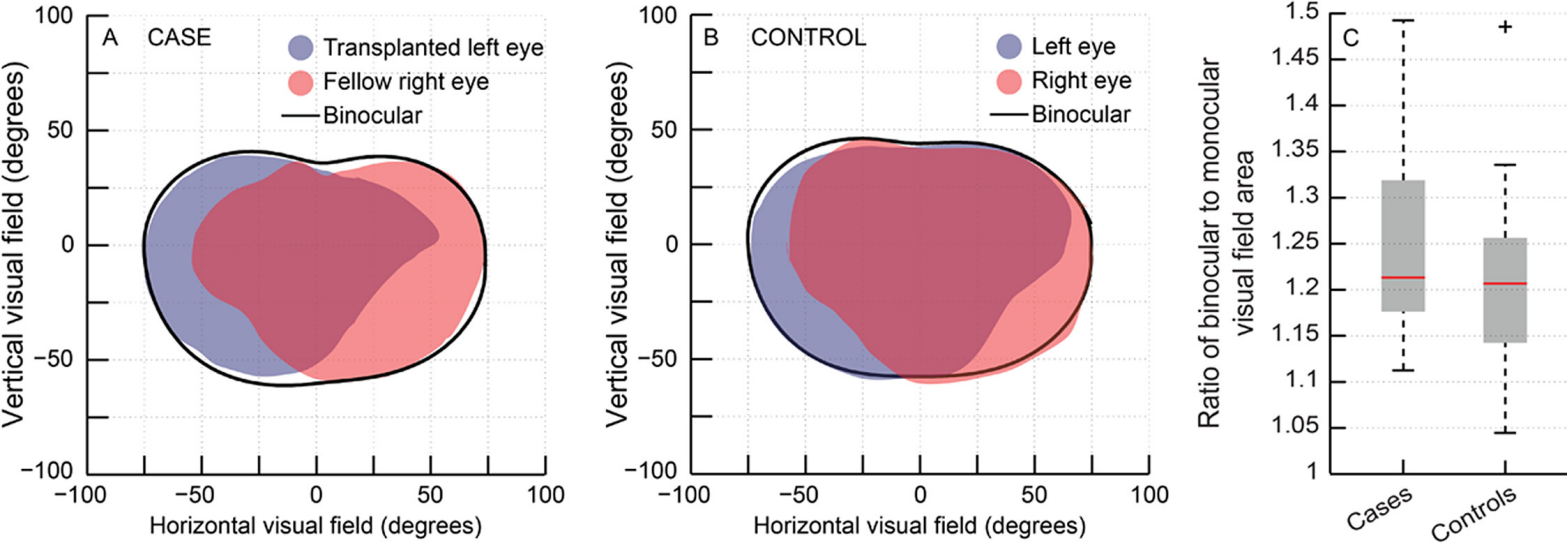
Binocular and monocular visual field of a representative case (panel A) and control (panel B) who participated in this study. Ratio of binocular to monocular visual field area in cases and controls (panel C). Details of Box and Whisker plot same as Fig 1.

The area occupied by the binocular visual field of both cases and controls was larger than the area occupied by the individual eye’s visual field (Fig 2A and 2B). Temporal crescents in the visual field were evident in the data of every subject that participated in the study (Fig 2A and 2B). The median ratio of binocular to monocular visual field area was 1.21 (1.18–1.29) in cases and 1.20 (1.14–1.25) in controls, indicating that there was a median increase in the binocular visual field area of 21% and 20% in cases and controls, respectively, vis-à-vis, their respective monocular visual field area (U = 127; n = 36; p = 0.3) (Fig 2C). The transplanted eye of cases indeed therefore contributed to an expansion of the binocular visual field, with the magnitude of expansion being very similar to those of healthy controls [33].

**Fig 3:**
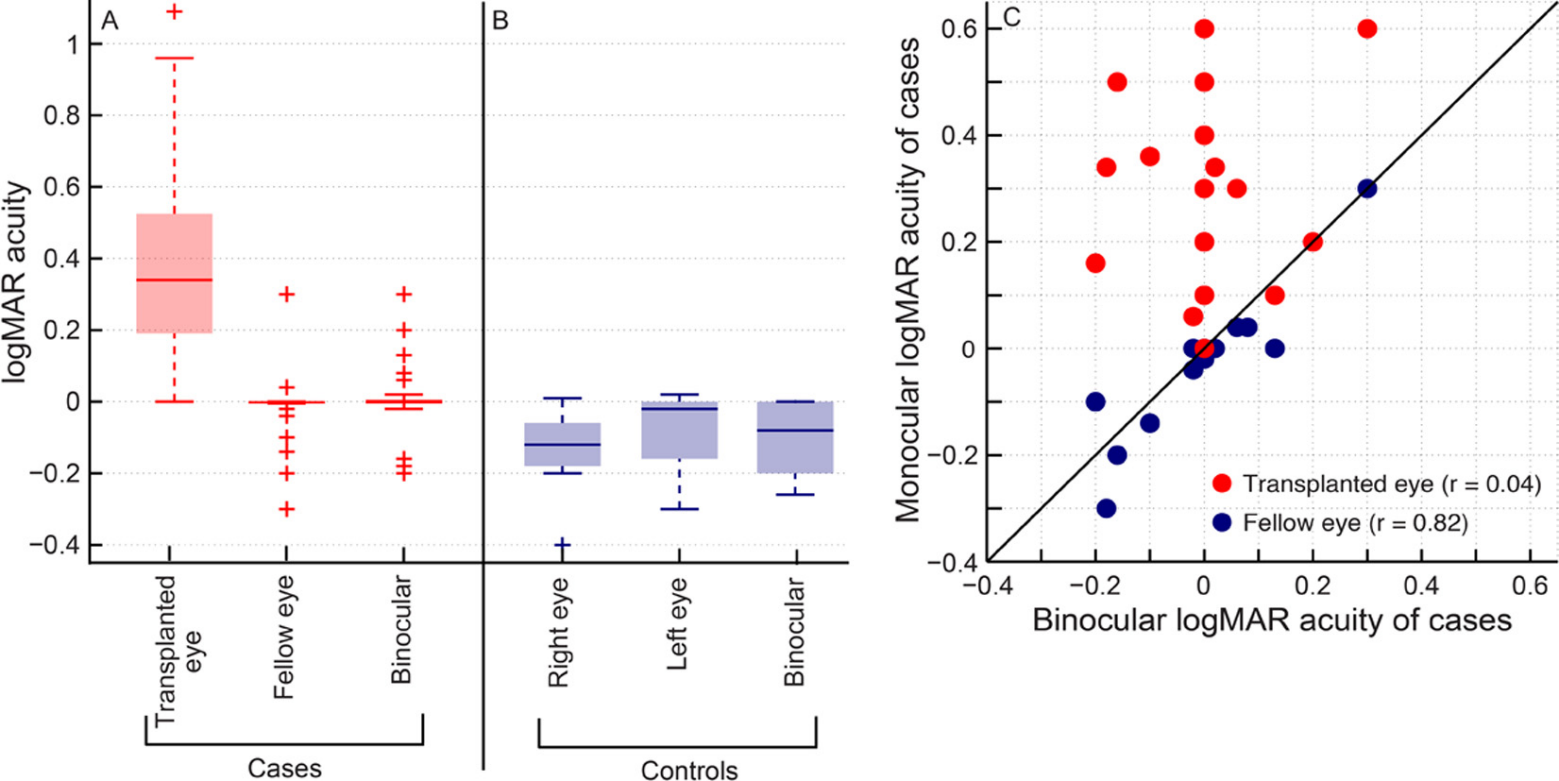
Monocular and binocular high-contrast logMAR acuity of cases (panel A) and controls (panel B) that participated in this study. Details of Box and Whisker plot same as Fig 1. Scatter diagram of monocular logMAR acuity of cases from the transplanted eye and the healthy fellow eye plotted against their corresponding binocular logMAR acuity (panel C). Correlation coefficient between binocular and the respective monocular logMAR acuities are shown in parenthesis in this panel.

Median best-spectacle corrected monocular logMAR acuity of the transplanted eye [0.34 (0.2–0.5)] was significantly poorer than that of the fellow eye [0.00 (0.00 –-0.02)] (U = 20.5; n = 50; p<0.001) and to that of controls [monocular: -0.12 (-0.18–0.00); binocular: -0.08 (-0.2–0.00)] (U = 14; n = 50; p<0.001) (Fig 3). Interocular difference in logMAR acuity of cases and controls was -0.3 (-0.6 –-0.2) and -0.04 (-0.12–0.00), respectively (U = 166; n = 50; p = 0.004). Binocular logMAR acuity of cases [0.00 (0.00–0.00)] was similar to the logMAR acuity of fellow eye (U = 282; n = 50; p = 0.5), with a Spearman’s rank correlation of 0.82 (p<0.001) (Fig 3A and 3C). Binocular logMAR acuity was poorly correlated with the transplanted eye’s acuity (r = 0.04; p = 0.83) (Fig 3C). Binocular spatial resolution of cases was therefore largely defined by the healthy eye’s resolution, with little influence of the transplanted eye. A multiple regression analysis indicated that, other than the best-corrected logMAR acuity of healthy fellow eye (p<0.001), other variables including the subject’s age, duration of the trauma, best corrected logMAR acuity and HORMS of transplanted eye, fellow eye and interocular difference in HORMS did not have any impact on the binocular acuity of cases (p>0.37).

**Fig 4:**
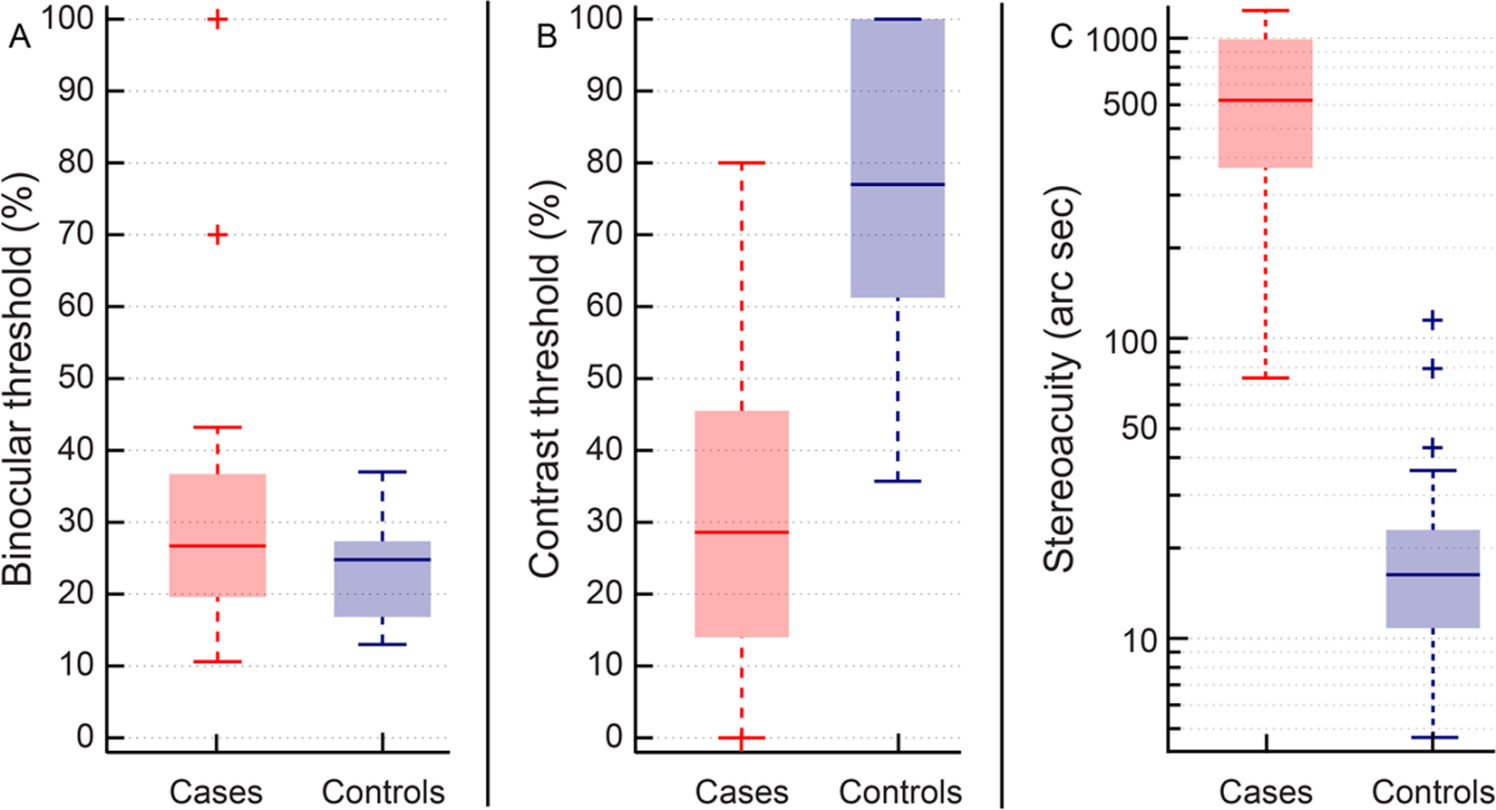
Binocular threshold (panel A), contrast threshold (panel B) and stereoacuity (panel C) of cases and controls that participated in this study. Details of Box and Whisker plot same as Fig 1.

The median binocular threshold was similar in both cases [26.7% (21.6–35.75%)] and controls [24.8% (17.2–27.2%)] (U = 189; n = 46; p = 0.16) (Fig 4A). This result is somewhat expected, for the signal and noise dots were both presented binocularly in this paradigm and, much like logMAR acuity, the binocular threshold may also be determined largely by the fellow eye of cases (Fig 4A). The median contrast threshold was significantly lower in cases [28.6% (14.5–45.2%)] than in controls [77% (62–100%)] (U = 42; n = 45; p<0.001) (Fig 4B), indicating that coherent motion perception could be abolished in cases with only 28.6% of median contrast in the healthy eye while it required up to 77% of median contrast in one eye to abolish coherent motion perception in controls. These results may be interpreted to indicate that the magnitude of suppression of the transplanted eye of cases was significantly greater than the magnitude of suppression of one random eye of controls.

The results of stereoacuity largely followed the expectations of the contrast threshold results, with the median stereoacuity of cases [620arc sec (370–988arc sec)] being significantly poorer than that of controls [16.3arc sec (11.3–22.4arc sec)] (U = 45.5; n = 50; p<0.001) (Fig 4C). Disparity-based depth detection was therefore significantly impaired following unilateral corneal transplantation, vis-à-vis, age-matched healthy controls. A second multiple regression analysis indicated that the patient’s age, duration of the trauma, best corrected HCVA, HORMS and the contrast and binocular thresholds did not significant impact on the stereoacuity of cases (p>0.31).

**Fig 5:**
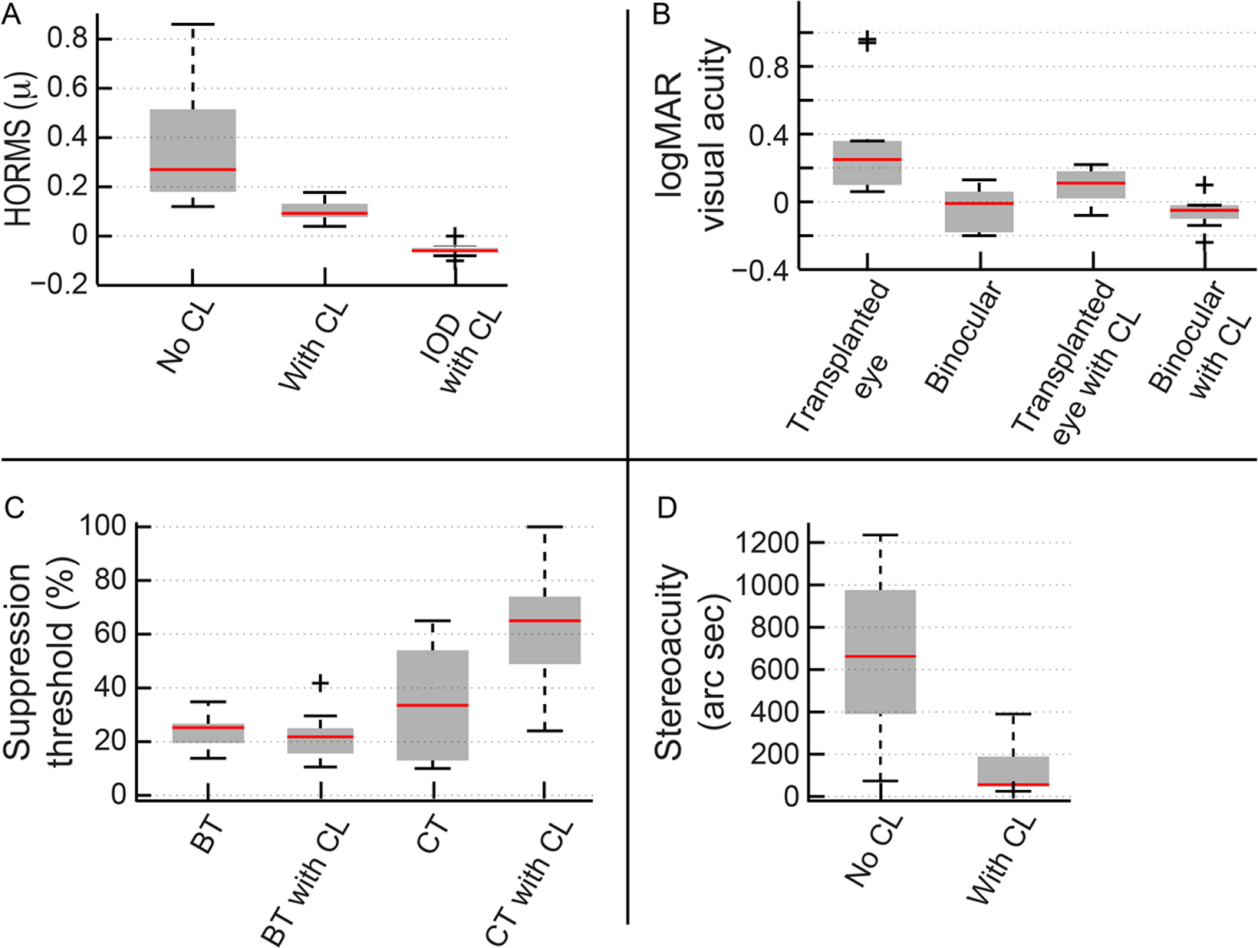
HORMS (panel A), high-contrast logMAR acuity (panel B), binocular thresholds (BT) and contrast thresholds (CT) (panel C) and stereoacuity (panel D) obtained with and without RGP contact lens in the second experiment of this study. Details of Box and Whisker plot same as Fig 1.

The median HORMS decreased significantly from 0.27μ (0.19–0.49μ) without contact lens to 0.09μ (0.08–0.12μ) with RGP contact lens wear (Wilcoxon Sign rank test Z = -2.52; n = 17; p = 0.01) (Fig 5A). The median interocular difference in HORMS also decreased from 0.22μ (0.13–0.42μ) without contact lens to 0.05μ (0.02–0.07μ) with contact lens wear (Z = -2.52; n = 17; p = 0.01) (Fig 5A). The median best-corrected logMAR acuity of the transplanted eye also improved from 0.25 (0.11–0.35) with spectacles to 0.11 (0.03–0.17) with contact lens wear (Z = -2.4; n = 20; p = 0.01). Median binocular logMAR acuity did not show any significant improvement [spectacles: -0.01(-0.14–0.04); contact lens: -0.05 (-0.09 –-0.02); p = 0.4], as binocular acuity appeared to be largely defined by the healthy eye’s acuity that was not manipulated during this experiment (Fig 5B). The contrast threshold of cases in the motion coherence task increased from 33.5% (13–53%) with spectacle wear to 65% (72–50%) with contact lens wear (Z = -2.1; n = 17; p = 0.02), even while there was no change in the binocular threshold values in these subjects [both: ~25.2% (21.7–26.35%)] (Fig 5C). The higher contrast required in the fellow eye to abolish coherent motion perception of the transplanted eye suggested that the magnitude of suppression in the latter decreased following contact lens wear. Median stereoacuity improved from 662.3arcsec (417.5–944.6arc sec) with spectacles to 56.6arc sec (47.68–181.6arc sec) with contact lenses (Z = -2.8; n = 20; p = 0.005) (Fig 5D).

## Discussion

The results of this study indicate that the transplanted eye of individuals undergoing penetrating keratoplasty for unilateral corneal disease participates in gross binocular visual functions (visual field expansion) but offers limited support to fine levels of binocularity (stereoacuity) (Figs 2–4). These individuals may therefore experience greater levels of peripheral visual awareness following surgery due to the expanded visual field but they may not benefit a great deal for binocular visual resolution or stereoacuity. These individuals may remain functionally monocular even after transplantation and may continue to use only their healthy fellow eye for all functional vision tasks like reading, watching television, etc. They may also rely more on monocular cues provided by their healthy fellow eye to obtain depth information as disparity processing is significantly impaired and the transplanted eye appears to be on the verge of suppression (Fig 4B and 4C). The median (25^th^–75^th^ IQR) follow-up duration between the corneal transplantation and the present investigation was 16.9 (9.2–33.3) months in all cases (Table 1), suggesting that the results reported here represent fairly well-established measures of visual performance and not a transient behavior when the eye was in a state of flux after surgery (e.g. during the corneal wound healing process).

The optical quality of the transplanted eye appears to play a crucial role in determining the extent of its participation in the habitual binocular viewing process. As observed in previous studies[12,13,14], the transplanted eye remained optically inferior to the healthy fellow eye because of greater magnitudes of uncorrected HOA’s (Fig 1). An improvement in the transplanted eye’s optical quality and a reduction in the difference in optical quality between the transplanted and healthy fellow eye produces a commensurate improvement in the fine levels of binocularity (Fig 5). The intersubject variability of HOA’s and stereoacuity also reduced with RGP contact lens wear, suggesting that the optical quality and visual performance became more homogenous when the aberrated donor cornea was replaced with a more uniform RGP contact lens surface (Fig 5A & 5D). As expected, gross tasks such a visual field expansion that were already up to mark with spectacles and monocular spatial vision tasks like logMAR acuity that were biased towards the healthy fellow eye remained unchanged with an improvement in the optics of the transplanted eye’s (Fig 5B). The metrics of evaluating the corneal transplantation procedure that are currently centered around the anatomical health and acuity of the transplanted eye can now be expanded to include measures of the transplanted eye’s optical quality and the level of binocularity restored in order to assess the functional benefit of transplantation in patients with unilateral corneal disease. Overall, physicians will have to pay attention to assessing and improving image quality in post transplant eyes in order to provide full benefit of binocular vision to the patient. The steps in this could include improved surgical technique to ensure superior corneal topography, postoperative modifications or use of corneal topography correcting contact lenses [4].

The results of logMAR visual acuity, suppression thresholds and stereoacuity obtained in this study are all somewhat expected from previous knowledge of how the visual system handles inter-ocular differences in image quality. Binocular logMAR visual acuity tends to follow the acuity of the least ametropic eye in the presence of induced spherical and astigmatic anisometropia [17,18,19]. The retinal image that is relatively more blurred is temporarily “suppressed” and visual resolution is biased towards the fellow eye [18,19,34]. This, in fact, forms the basis of monovision contact lens correction in presbyopia wherein anisometropia is intentionally induced by wearing a myopic soft contact lens over the non-dominant eye for focusing at near while the dominant eye is corrected for distance vision [35]. Despite the persistence of interocular difference in image quality due to HOA’s (Fig 1C), binocular logMAR acuity of cases remained more or less equal to the acuity of their healthy fellow eye (Fig 2). The input provided by the transplanted eye may be “suppressed” much like the monovision scenario and binocular spatial resolution may be optimized by increasing weightage to the healthy fellow eye’s input. The lower contrast thresholds of cases, vis-à-vis, controls reflect the increased magnitude of suppression of the transplanted eye in the current study (Fig 4B).

Tasks requiring the matching of corresponding features in the two eyes (e.g. stereoacuity) tend to deteriorate in the presence of interocular difference in image quality [15,17,36]. Poor stereoacuity of cases with their best-corrected spectacles and its improvement with RGP contact lens are very much in line with the existing literature (Figs 4C and 5D). Interestingly, HORMS did not have a significant influence on the stereoacuity of cases in this study. This may be because of a ceiling effect on stereoacuity caused by the high magnitude of HORMS in the transplanted eye (median: 0.35μ; median interocular difference in HORMS: 0.25μ) (Fig 1). Indeed, the deterioration of other binocular functions like contrast summation and disparity detectability saturate for interocular difference in HORMS >0.25μ [16,37]. Alternatively, the HORMS per se may not have a direct bearing on stereoacuity but its manifestation in terms of contrast loss or phase shifts in the retinal image may have a greater influence on stereoacuity [38]. It is also possible that any aniseikonia (interocular difference in retinal image size) that may be induced by spectacle wear in the transplanted eye may have caused the stereoacuity loss [15,30,36]. HORMS of the transplanted eye, interocular difference in HORMS, the associated contrast loss and phase shifts in the retinal image, interocular difference in logMAR acuity and aniseikonia all would have reduced with RGP contact lens wear leading to a commensurate improvement in stereoacuity of these individuals (Fig 5D). A systematic analysis of the impact of these parameters on stereoacuity is however beyond the scope of the current study.

This study has three limitations. First, functional participation of the transplanted eye was assessed only post-operatively and, therefore, the status of binocularity prior to surgery remains unknown. Given the poor pre-operative visual acuity of these subjects, it is likely that the pre-operative contribution of the affected eye to binocular visual functions was, at best, gross. The exact change in binocular visual performance after transplantation may only be obtained when the pre- and post-operative data of the same subject are compared relative to each other. Second, the sample size of cases and controls in this study was relatively small (n = 25) and the cases were selectively chosen to have corneal transplantation as the only ocular intervention and not associated co-morbidities. This combined with the relatively large inter-subject variability in the results (see error bars in Figs 1–4) preclude us from generalizing the results to the entire pool of patients undergoing transplantation for unilateral corneal disease. The results shown here are however still very valid and informative for the controlled cohort of cases selected for this study. Third, this study was only concerned with the visual functions of individuals undergoing corneal transplantation. Other important outcome measures of treatment such as the post-operative quality of life [7,8], cost-utility ratio of treatment [39], supply-demand ratio of donor tissue [3] and the risk of morbidity with unilateral vision [40] were all not evaluated. A comprehensive analysis of all these variables is necessary to make a final judgment on the utility of the transplantation procedure in individuals with unilateral corneal disease.
